# The antimicrobial capacity of *Cistus salviifolius* and *Punica granatum* plant extracts against clinical pathogens is related to their polyphenolic composition

**DOI:** 10.1038/s41598-020-80003-y

**Published:** 2021-01-12

**Authors:** Francisco Javier Álvarez-Martínez, Juan Carlos Rodríguez, Fernando Borrás-Rocher, Enrique Barrajón-Catalán, Vicente Micol

**Affiliations:** 1 Instituto de Biología Molecular y Celular (IBMC) and Instituto de Investigación, Desarrollo e Innovación en Biotecnología Sanitaria de Elche (IDiBE), Universidad Miguel Hernández (UMH), 03202 Elche, Spain; 2 Microbiology Section, University General Hospital of Alicante, Alicante Institute for Health and Biomedical Research (ISABIAL Foundation, Alicante, Spain; 3 Statistics and Operative Research Department, Miguel Hernández University (UMH), Avda. Universidad s/n, 03202 Elche, Spain; 4 CIBER, Fisiopatología de la Obesidad y la Nutrición, CIBERobn, Instituto de Salud Carlos III (CB12/03/30038), Madrid, Spain

**Keywords:** Microbiology, Antimicrobials, Biotechnology, Applied immunology, Drug discovery

## Abstract

Antimicrobial resistance poses a serious threat to human health worldwide. Plant compounds may help to overcome antibiotic resistance due to their potential resistance modifying capacity. Several botanical extracts and pure polyphenolic compounds were screened against a panel of eleven bacterial isolates with clinical relevance. The two best performing agents, *Cistus salviifolius* (CS) and *Punica granatum* (GP) extracts, were tested against 100 *Staphylococcus aureus* clinical isolates, which resulted in average MIC_50_ values ranging between 50–80 µg/mL. CS extract, containing hydrolyzable tannins and flavonoids such as myricetin and quercetin derivatives, demonstrated higher activity against methicillin-resistant *S. aureus* isolates. GP extract, which contained mostly hydrolyzable tannins, such as punicalin and punicalagin, was more effective against methicillin-sensitive *S. aureus* isolates. Generalized linear model regression and multiple correspondence statistical analysis revealed a correlation between a higher susceptibility to CS extract with bacterial resistance to beta-lactam antibiotics and quinolones. On the contrary, susceptibility to GP extract was related with bacteria sensitive to quinolones and oxacillin. Bacterial susceptibility to GP and CS extracts was linked to a resistance profile based on cell wall disruption mechanism. In conclusion, a differential antibacterial activity against *S. aureus* isolates was observed depending on antibiotic resistance profile of isolates and extract polyphenolic composition, which may lead to development of combinatorial therapies including antibiotics and botanical extracts.

## Introduction

**Figure 1:**
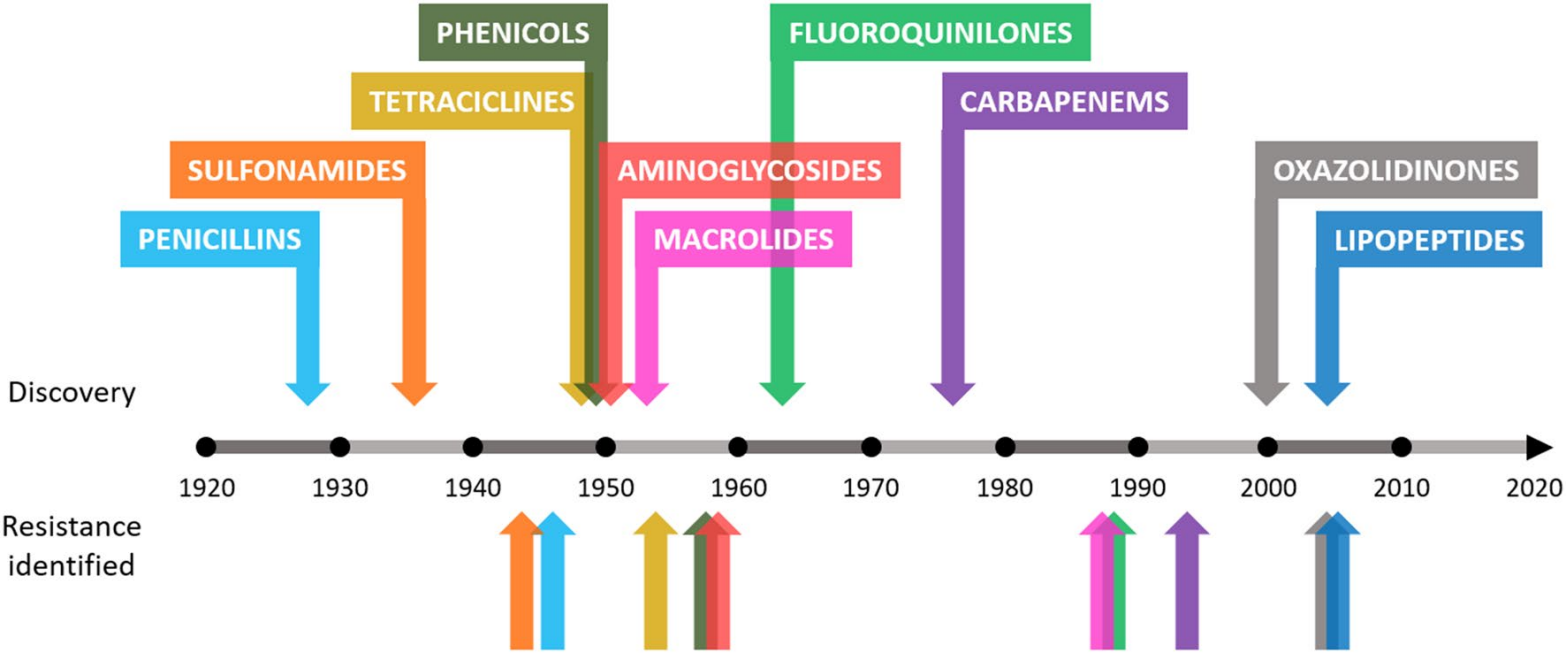
Approximate dates of discovery of new classes of antibiotics and identification of bacterial resistance.

The increasing number of multidrug-resistant microorganisms represents a serious threat to human health worldwide, and unless drastic measures are taken, this number will continue to increase. There is an imminent risk of having very few therapeutic alternatives in the management of some serious infectious processes. The prognosis for the year 2050 is 10 million deaths each year and a global economic cost of 86 trillion dollars derived from antibiotic resistance^1^. Moreover, few new antibiotics are being discovered by the scientific community at present and in recent years. Pharmaceutical companies are uncertain when investing in the development of new antibiotics due to the possibility of rapid bacterial resistance development, resulting in an inability to recover their investment^2^. This concerning trend can be observed in Fig. 1.

As traditional drug therapies are losing efficacy, novel therapies based on natural antimicrobial compounds are emerging as alternative or complementary treatments against nosocomial infections. Natural combinations such as plant extracts containing a wide range of different molecules, including polyphenols, have demonstrated antimicrobial activity. These combinations can act against many different bacterial molecular targets, sometimes potentially avoiding common antibiotic resistance mechanisms^3^. There is evidence of polyphenols and plant extracts capable of disrupting the bacterial plasma membrane, inhibiting efflux pumps, inhibiting the formation of biofilms and inhibiting the action of proteins related to antimicrobial resistance such as PBP2a^4^.

Polyphenols are compounds with large structural variability but common phenolic moieties in their structure (Supplementary Fig. S1). In addition, polyphenols usually appear as conjugated forms with carbohydrates or form esters with organic acids, contributing to a considerable increase in their chemical diversity. Polyphenolic compounds have modulated their diversity throughout evolution to act as ligands of many different molecular targets, generating high molecular promiscuity^5^. This multitarget trait is key in the antimicrobial capacity of plant polyphenols and in their synergistic effects with traditional antibiotics^6^. Examples of polyphenols with demonstrated antimicrobial capacity are the flavonols quercetin and kaempferol, with reported MIC values as low as 1.95 µg/mL and 7.8 µg/mL respectively, against *S. aureus*. Botanical extracts rich in polyphenols have also been shown to possess significant antibacterial capacity. For instance, extracts obtained from *Cistus ladanifer*, *Cistus albidus*, *Cistus clusii* and *Cistus salviifolius* have shown MIC values under 100 µg/mL against *S. aureus*^3^. The chemical composition of botanical extracts derived from different *Cistus* species and that of *P. granatum* have been fully reviewed in the past^7,8^. Moreover, the relationship between the antimicrobial activity against *S. aureus* and *Escherichia coli* and the presence in these extracts of hydrolyzable tannins and flavonoids has been previously reported^9–11^.

The objective of the present work was to test the antimicrobial capacity of complex botanical extracts, such as *P. granatum* and *C. salviifolius* with previously reported antimicrobial capacity, and some selected pure polyphenolic compounds to make a selection of the best performing ones for further resistant bacteria profiling. In addition, it was intended to explore whether a relationship existed between the antimicrobial activity of the selected extracts and the antibiotic resistance profile of a panel of clinical isolates of *S. aureus.* Moreover, by using different statistical approaches, we also investigated if the polyphenolic composition or the type of extract might play a role in the bacterial susceptibility, according to the antibiotic resistance profile and mechanism involved in the resistance.

## Results

### First screening: disk diffusion assay

A panel consisting of 3 different plant extracts (*CS, C. salviifolius*; PN, *Citrus paradisi*; GP, *P. granatum*) and 7 pure polyphenolic compounds (GA, gallic acid; P, punicalagin; Q3G, quercetin-3-glucuronide; M, myricetin; N, naringenin; EA, ellagic acid) was initially selected for the first antimicrobial screening based on existing literature and previous experiments of the research group in which these agents showed activity against bacterial models^9,12^. The screening was performed using various clinical isolates of the following microbial species: *Staphylococcus aureus*, *Enterococcus faecalis*, *Enterococcus faecium*, *Escherichia coli*, *Klebsiella pneumoniae*, *Enterobacter *spp., *Serratia marcescens*, *Salmonella *spp., *Pseudomonas aeruginosa*, *Acinetobacter baumannii* and *Stenotrophomonas maltophilia*.

A disk diffusion antimicrobial assay was performed as the first screening to choose the most active compounds or extracts for further exhaustive antibiograms. The percentage of sensitive isolates of each bacterial species for each extract or pure compound is displayed in Table 1. The isolate that had an inhibition halo for a given compound was considered a susceptible isolate to that compound, no matter its diameter. Differences in the number of clinical isolates used for each bacterial species were due to the variability in hospital bacterial collection derived from patients during the selection period of 30 days.

**Table 1:**
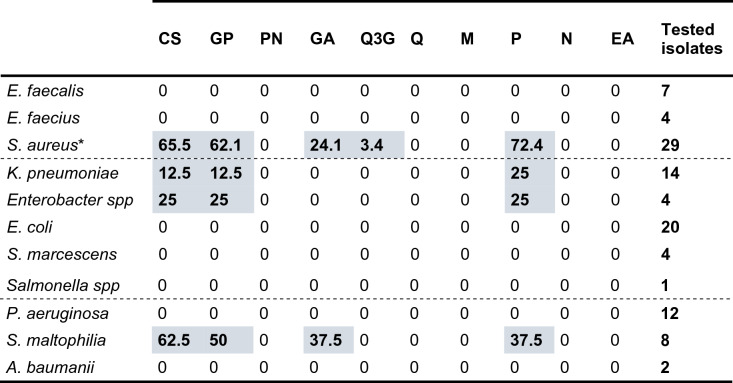
Percentage of sensitive isolates of gram-positive cocci (rows 1–3), Enterobacteria (rows 4–8) and nonfermentative Gram-negative bacilli (rows 9–11) species to each extract or pure compound. The dotted lines separate the different groups of bacteria. *S. aureus includes both MRSA and MSSA isolates indistinctly.

Based on the obtained results, further investigation was conducted to determine the activity of the most effective agents in this first screening against the most sensitive species of clinical relevance. According to this reasoning, CS and GP were chosen for use against *S. aureus* due to their crucial clinical importance, ease of laboratory culture and good results obtained in the first screening. Although compound P (pure punicalagin) was also effective against several bacteria, it was rejected for further tests due to its high economic cost and because it was the main component of the GP and CS extracts, so its activity would be somewhat covered by these extracts.

### Phenolic content and HPLC–MS molecular characterization of the extracts

After the first screening, the total phenolic content of the selected extracts was then measured by using the gallic acid equivalence (GAE) method and their fully composition were characterized by HPLC–MS as described in the following section. The CS extract showed a total phenolic content of 60.1 ± 5.5 g GAE/100 g extract. In contrast, the GP extract showed a lower total phenolic content with 35.9 ± 2.5 g GAE/100 g extract.

**Figure 2:**
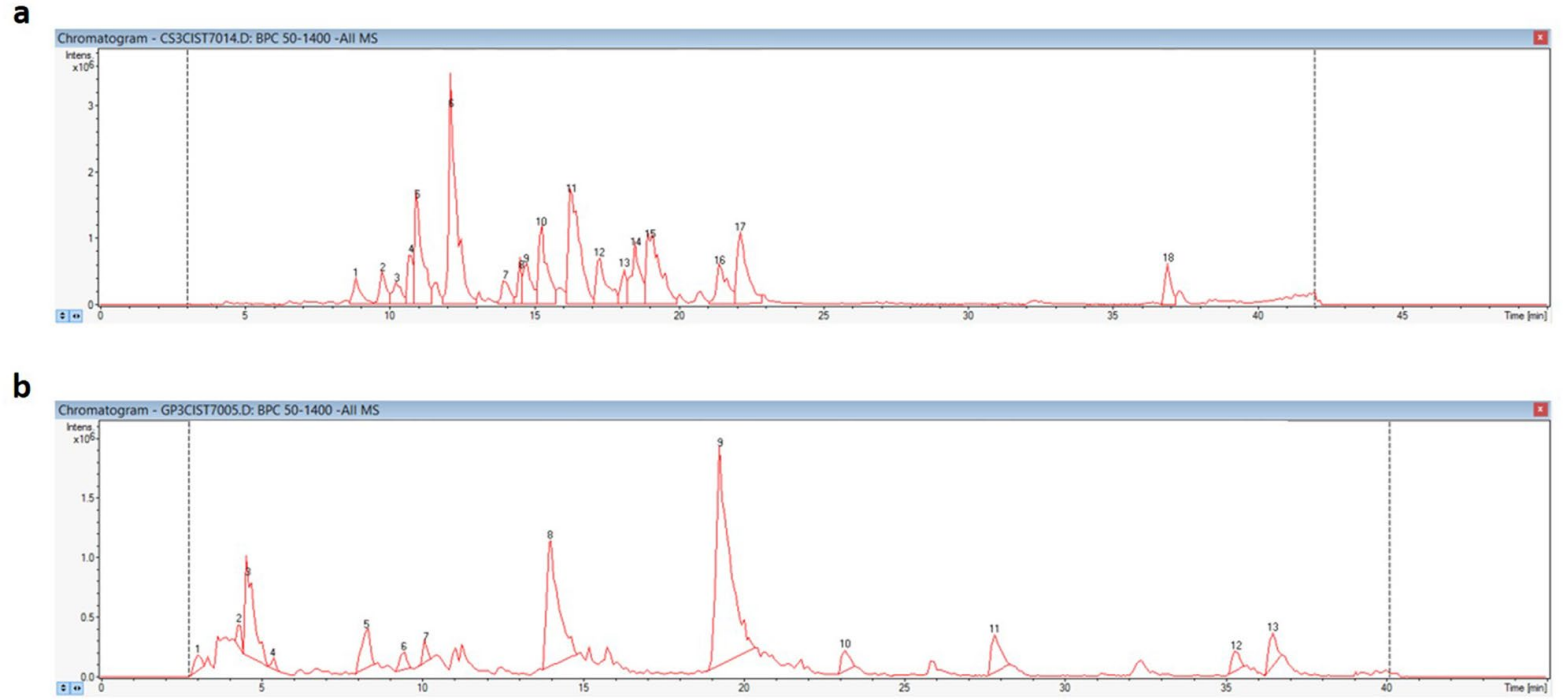
Base peak chromatograms of the CS (**a**) and GP extract (**b**) obtained by HPLC analysis.

The CS and GP extracts were characterized using HPLC–MS using methods specifically designed for these botanical extracts as described in the Materials and Methods section. Base peak chromatograms are shown in Fig. 2, and the identified compounds and chromatographic and mass spectral data are included in Table 2 for CS and Table 3 for GP extracts. The quantification of the main compounds in the extracts was done by construction of standard curves of punicalagin and quercetin as representative compounds for hydrolyzable tannins and flavonoids respectively. Standard curves for both compounds are included in Supplementary Fig. S2.

**Table 2:** Identified compounds in the CS extract using HPLC–MS.

Peak	RT (min)	Relative area (%)	[M−H]^−^	MS/MS	Proposed compound	References
1	8.8	2.101	609	423, 441	(–)-(Epi)gallocatechin-(epi)gallocatechin dimer	^21^
2	9.8	2.243	783	275, 301, 451, 481	Pedunculagin I	^22^
3	10.3	2.036	337	161	Coumaroylquinic acid	^23^
4	10.7	3.022	761	423, 609	Prodelphinidin B2-3′-*O*-gallate	^12^
5	10.9	8.626	1083	301, 601, 781	Punicalagin isomer I	^12^
6	12.1	17.557	1083	301, 601, 781	Punicalagin isomer II	^12^
7	14.0	1.941	343	181	*Not identified*	
8	14.5	1.807	341	179	Caffeoyl-hexose	^21^
9	14.7	3.548	453	151, 169, 313	Ligstroside derivate	^24^
10	15.2	6.656	631	317, 479	*Not identified*	
11	16.3	13.326	479	179, 271, 316	Myricetin hexoside	^21^
12	17.2	5.055	615	301	Dehydrated tergallic-C-glucoside	^23^
13	18.1	1.824	449	179, 271, 316	Myricetin 3-arabinoside isomer I	^12^
14	18.5	5.761	449	179, 271, 316	Myricetin 3-arabinoside isomer II	^12^
15	19.0	10.097	463	151, 301	Quercetin glucoside	^12^
16	21.4	4.174	433	301	Ellagic acid-7-xiloside isomer I	^25^
17	22.1	7.676	433	301	Ellagic acid-7-xiloside isomer II	^25^
18	36.9	2.549	593	285, 447	Kaempferol diglycoside	^12^

**Table 3:** Identified compounds in the GP extract using HPLC–MS.

Peak	RT (min)	Relative Area (%)	[M−H]^−^	MS/MS	Proposed compound	References
1	3.0	1.531	193	149	*Not identified*	
2	4.3	1.659	481	275, 301	HHDP glucoside isomer	^26^
3	4.6	12.315	481	275, 301	HHDP glucoside isomer II	^26^
4	5.4	0.712	331	169, 271	Galloyl glucose	^23^
5	8.3	4.932	781	271, 601, 721	Punicalin	^27^
6	9.4	1.674	783	275, 301, 481	Pedunculagin I	^22^
7	10.1	1.377	1083	301, 601, 781	Punicalagin isomer I	^12^
8	14.0	21.511	1083	301, 601, 781	Punicalagin isomer II	^12^
9	19.2	40.990	1083	301, 601, 781	Punicalagin isomer III	^12^
10	23.1	2.411	801	347, 649	Punigluconin	^22^
11	27.8	4.977	463	301	Quercetin glucoside	^12^
12	35.3	2.247	447	300	Ellagic acid rhamnoside	^28^
13	36.4	3.662	301	229, 257	Ellagic acid	^25^

The hydrolyzable tannins identified in the CS extract and quantified using the punicalagin standard were punicalagin (peaks 5 and 6 as isomers I and II respectively) and pedunculagin (peak 2). These compounds exhibited concentrations of 0.299 and 0.608 and 0.077 mg/mL punicalagin equivalents respectively. The total hydrolyzable tannin concentration in the CS extract was 0.984 mg/mL punicalagin equivalents, corresponding to 32.800% w/w of the extract. The hydrolyzable tannins identified in the GP extract were punicalagin (peaks 7, 8 and 9), punicalin (peak 5), pedunculagin I (peak 6) and punigluconin (peak 10). The determined concentrations of these compounds were 0.017, 0.270, 0.514, 0.062, 0.021 and 0.030 mg/mL punicalagin equivalents, respectively. The total hydrolyzable tannin concentration in the GP extract was 0.914 mg/mL punicalagin equivalents, corresponding to 30.460% w/w of the extract.

Quercetin was used as the representative flavonoid to quantify this polyphenolic compound group. The flavonoids identified in the CS extract were myricetin hexoside (peak 11), myricetin 3-arabinoside isomer I (peak 13), myricetin 3-arabinoside isomer II (peak 14), quercetin glucoside (peak 15) and kaempferol diglycoside (peak 18). The determined concentrations for these compounds were 0.0154, 0.0021, 0.0066, 0.0116 and 0.0029 mg/mL quercetin equivalents, respectively. The total flavonoid concentration in the CS extract was 0.0386 mg/mL quercetin equivalents, corresponding to 1.286% w/w of the extract. There was only one flavonoid identified in the GP extract, being quercetin glucoside (peak 11) with a determined concentration of 0.0057 mg/mL which corresponds to 0.190% w/w of the extract, approximately 10 times less than CS flavonoid content. The structures of the main compounds on each extract are shown in Supplementary Fig. S3 online.

### Second screening: antimicrobial assays using the microdilution method

**Figure 3:**
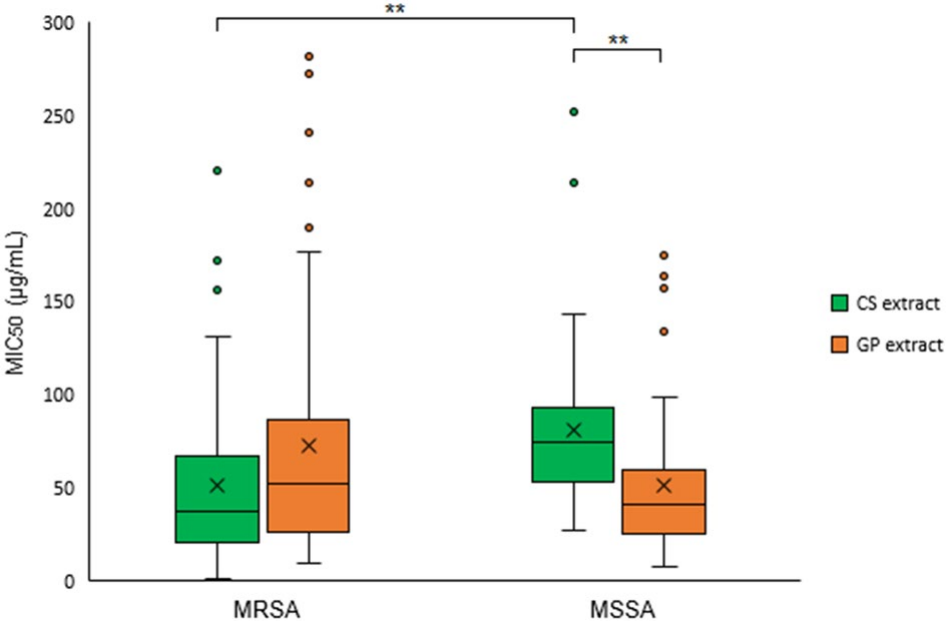
Box charts of the MIC_50_ distribution of the CS extract (green) and GP extract (orange) against MRSA and MSSA. The X within the box indicates the value of the mean, and the horizontal line indicates the median.

Microdilution in the p96 plate method was used for the second screening in which the antimicrobial activity of CS and GP extracts against 100 *S. aureus* clinical isolates (50 MRSA and 50 MSSA) was studied. Values for the minimum concentration that inhibit the bacterial growth by 50% (MIC_50_) were obtained for every single isolate as explained in methods section. The distribution of the MIC_50_ values of the two extracts against each of the 100 tested isolates of *S. aureus* is shown in Fig. 3. The specific MIC_50_ values for each extract can be seen in Supplementary Tables S1 and S2 online.

The mean MIC_50_ value of the CS extract against MRSA was 51.21 μg/mL, while that of the GP extract was 72.89 μg/mL. No significant differences between the MIC_50_ values of the two extracts were observed in this case (two-tailed *p* value = 0.0657 > 0.05). The average MIC_50_ value against MSSA of the CS extract was 80.70 µg/mL, while that of the GP extract was 51.67 µg/mL. There was a significant difference in the antimicrobial activity against MSSA between the two extracts (two-tailed *p* value = 0.0012 < 0.01, **).

On the contrary, when the activity of each extract against MRSA and MSSA isolates was compared, we encountered significant differences in relation to CS extract, which was more effective against MRSA (two-tailed *p* value = 0.0019 < 0.01, **) than against MSSA. No significant differences were found when comparing the activity of the GP extract against both MRSA and MSSA (two-tailed *p* value = 0.0709 > 0.05). The MIC_50_ values were independent for each isolate, and there was no direct correlation between CS and GP MIC_50_ values for the studied isolates (data not shown).

### Antibiotic resistance and extract activity relationship study

All isolates of *S. aureus* were characterized by their profiles of resistance to various antibiotics commonly used clinically to determine whether there was a relationship between strain resistance to antibiotics and the antimicrobial action of the extracts through their MIC_50_ values. These profiles are included for each single isolate in Supplementary Table S3 online.

**Figure 4:**
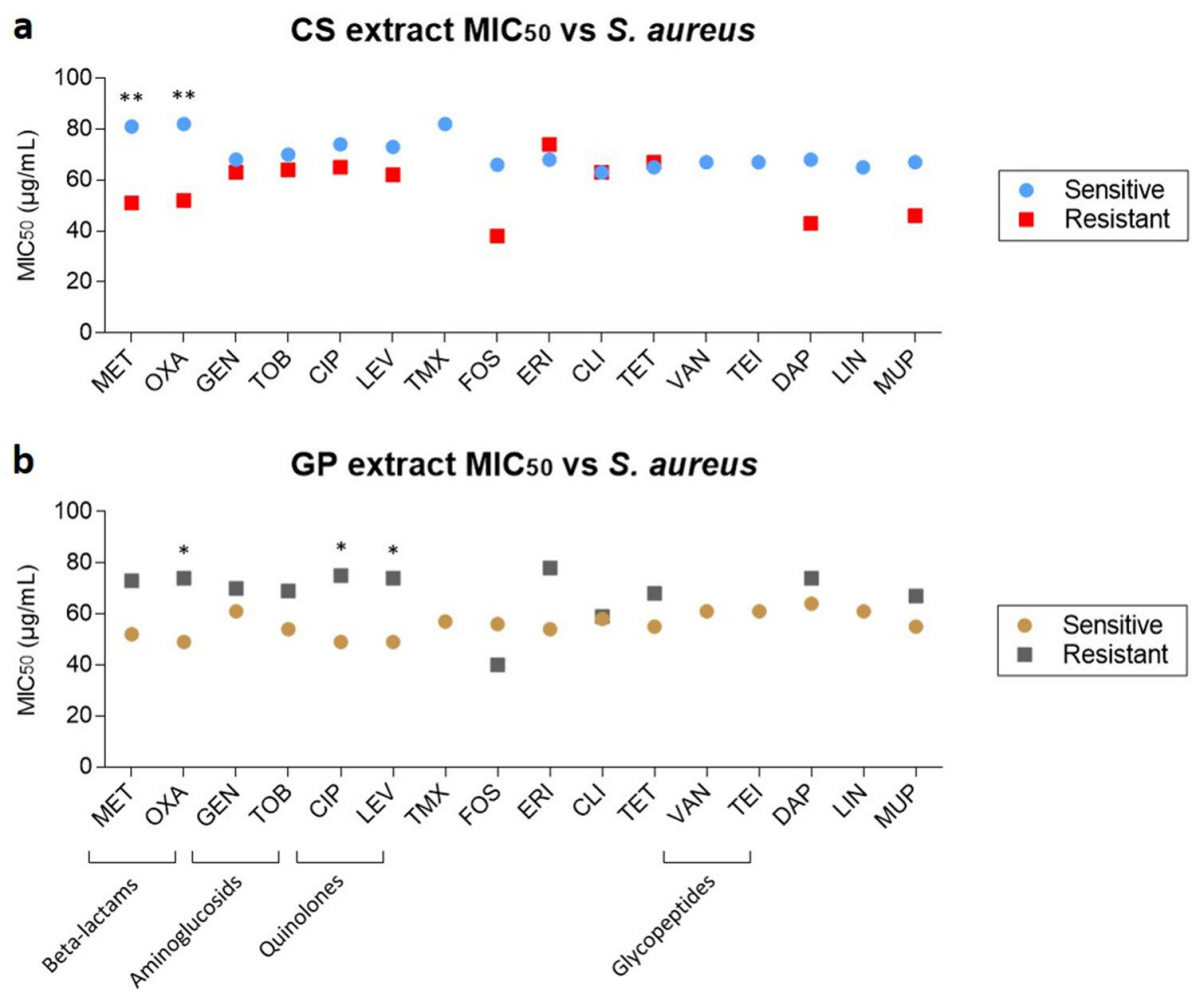
Comparative MIC_50_ values of the CS extract (**a**) and GP (**b**) extracts against S. aureus isolates grouped by antibiotic sensitivity (circles) or resistance (squares). *MET* methicillin, *OXA* oxacillin, *GEN* gentamicin, *TOB* tobramycin, *CIP* ciprofloxacin, *LEV* levofloxacin, *TMX* trimethoprim/sulfamethoxazole, *FOS* fosfomycin, *ERY* erythromycin, *CLI* clindamycin, *TET* tetracycline, *VAN* vancomycin, *TEI* teicoplanin, *DAP* daptomycin, *LIN* linezolid, *MUP* mupirocin. Error bars are not included in this figure to minimize the symbols included on it but are included in Supplementary Fig. S4 online.

In Fig. 4, the mean MIC_50_ values of the CS (a) and GP (b) extracts are represented for every sensitive or resistant isolate of *S. aureus*. Unlike the previous Fig. 3, now mean MIC_50_ values were grouped based on the resistance or sensitivity of the *S. aureus* isolates against the panel of clinical antibiotics previously used to characterize those isolates (see Supplementary Table S3). Individual comparisons for each antibiotic are also shown in Supplementary Fig. S4 online.

When grouped MIC values of CS and GP extracts for *S. aureus* isolates were analyzed by resistance to particular antibiotics, it could be observed that significant differences between certain groups of treatments existed depending on the antibiotic. The CS extract was generally more active against resistant bacteria than against sensitive ones (Fig. 4a). On the contrary, GP extract was more active against sensitive bacteria than against resistant ones (Fig. 4b) (red squares are located below blue circles but grey squares are above golden circles regardless of the resistance). CS extract showed a significantly higher antimicrobial capacity against *S. aureus* isolates that were resistant to beta-lactam antibiotics (methicillin and oxacillin), than that against sensitive isolates (*p* < 0.01, **). Similar results were obtained for other antibiotics, such as fosfomycin, daptomycin and mupirocin, but without showing statistical significance (*p* > 0.05). In contrast, the GP extract showed greater activity against sensitive isolates compared to resistant ones regardless the type of antibiotic (circles are below squares), with the single exception of fosfomycin. These differences were statistically significant (*p* < 0.05, *) for oxacillin and quinolones (ciprofloxacin and levofloxacin).

### Generalized linear model regression (GLMR)

GLMR was used to give a consistent explanation of the behavior of the MIC_50_ values of the CS and GP extracts against *S. aureus* isolates based on their profile of resistance to clinical antibiotics overall. Supplementary Tables S4 and S5 online show the results of the GLMR analysis, obtained as described in the methods section.

The results indicate that it was possible to predict the MIC_50_ value of the CS extract based on the resistance or sensitivity of each isolate to the antibiotics methicillin (*p* = 0.014, *), oxacillin (*p* = 0.014, *), ciprofloxacin (*p* = 0.003, **) and levofloxacin (*p* = 0.007, **). In contrast, this did not seem to be possible for the GP extract, since no parameter could significantly explain its contribution to the MIC_50_ value. In both cases, sensitivity to teicoplanin appeared to be significant, but this was because no isolates were found to be resistant to teicoplanin, making it impossible to compare them in the analysis and yielding values exactly equal to the value of the initial interception.

### Multiple correspondence analysis (MCA)

MCA was conducted to further determine the relationship among the antimicrobial activity of extracts and the susceptibility to a given antibiotic for each bacterial isolate. In MCA, relations between row and column variables and relations between different levels of each variable can both be obtained. This analysis allowed for categorical/categorized data to be transformed into cross tables and two-dimensional images of data, simplifying their interpretation^13^. Based on these criteria, two different solutions were explored with two MCA dimensions.

**Figure 5:**
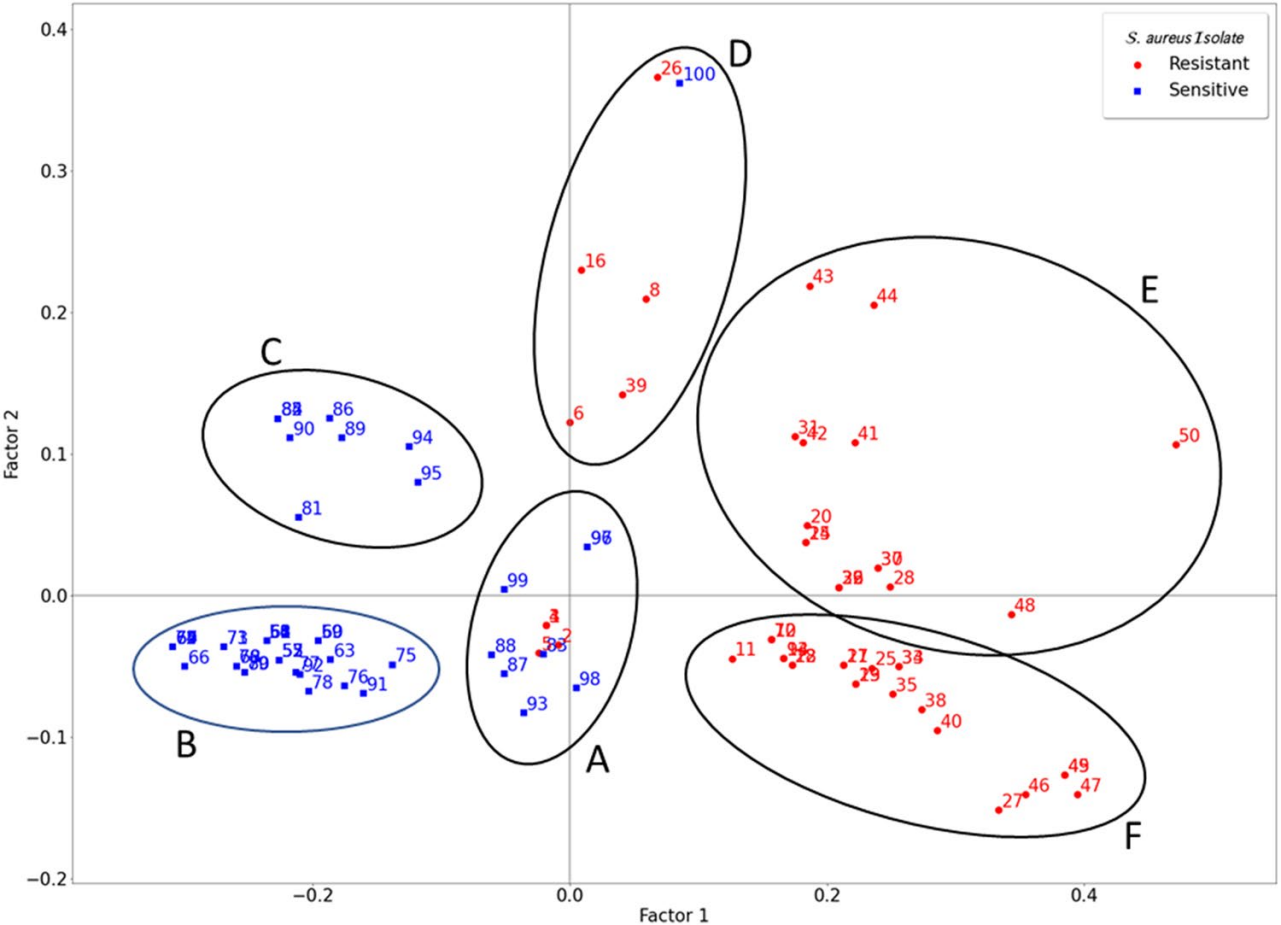
MCA results for individual isolates as points. Distribution of the different clinical isolates of *S. aureus* based on their profiles of resistance to antibiotics for clinical use and extracts.

The first solution was based on a direct representation of individual isolates as points. According to this approach, the first dimension accounted for 38.73% of the variance, and the second accounted for 7.22%, yielding a total of 45.94% of the variance being accounted for. The results are shown in Fig. 5.

As seen in this figure, there is a clear grouping of the MRSA isolates, with positive factor 1 values (red circles), while the MSSA group is mainly negative for this factor (blue squares). This grouping indicates that the different isolates behaved differently based on their methicillin resistance or sensitivity, confirming the results obtained in the previous sections in which the resistance against methicillin was determinant of the extracts’ activity (see Figs. 3 and 4). Furthermore, additional groupings can be observed in Fig. 5 (black capital letters). The A group contains a series of heterogeneous strains with resistances of up to four antibiotics, but without common characteristics, because it is close to the origin point. The B group contains 32 isolates mainly sensitive to most of the tested antibiotics, so it can be considered the most sensitive population of the study. The C group contained 9 isolates also presenting sensitivity to most of the antibiotics in group B, but 8 of them showed resistance to ERI and CLI antibiotics, whereas only intermediate resistance was obtained for some of the isolates contained in the B group. The D group contained only 6 isolates; up to 5 of them share resistance to beta-lactams (MET and OXA) and total or intermediate sensitivity to quinolones (CIP and LEV). Group E includes 15 isolates that have common resistance against beta-lactams and quinolones, accompanied by ERY resistance in 14 of them. Finally, the F group contained 25 isolates with resistance against beta-lactams and quinolones, but 24 isolates did not have resistance against ERY. Additionally, the F group contained most of the isolates with relatively low MIC_50_ values for the CS extract, with 17 of the isolates among the 50 most sensitive isolates to the CS extract.

**Figure 6:**
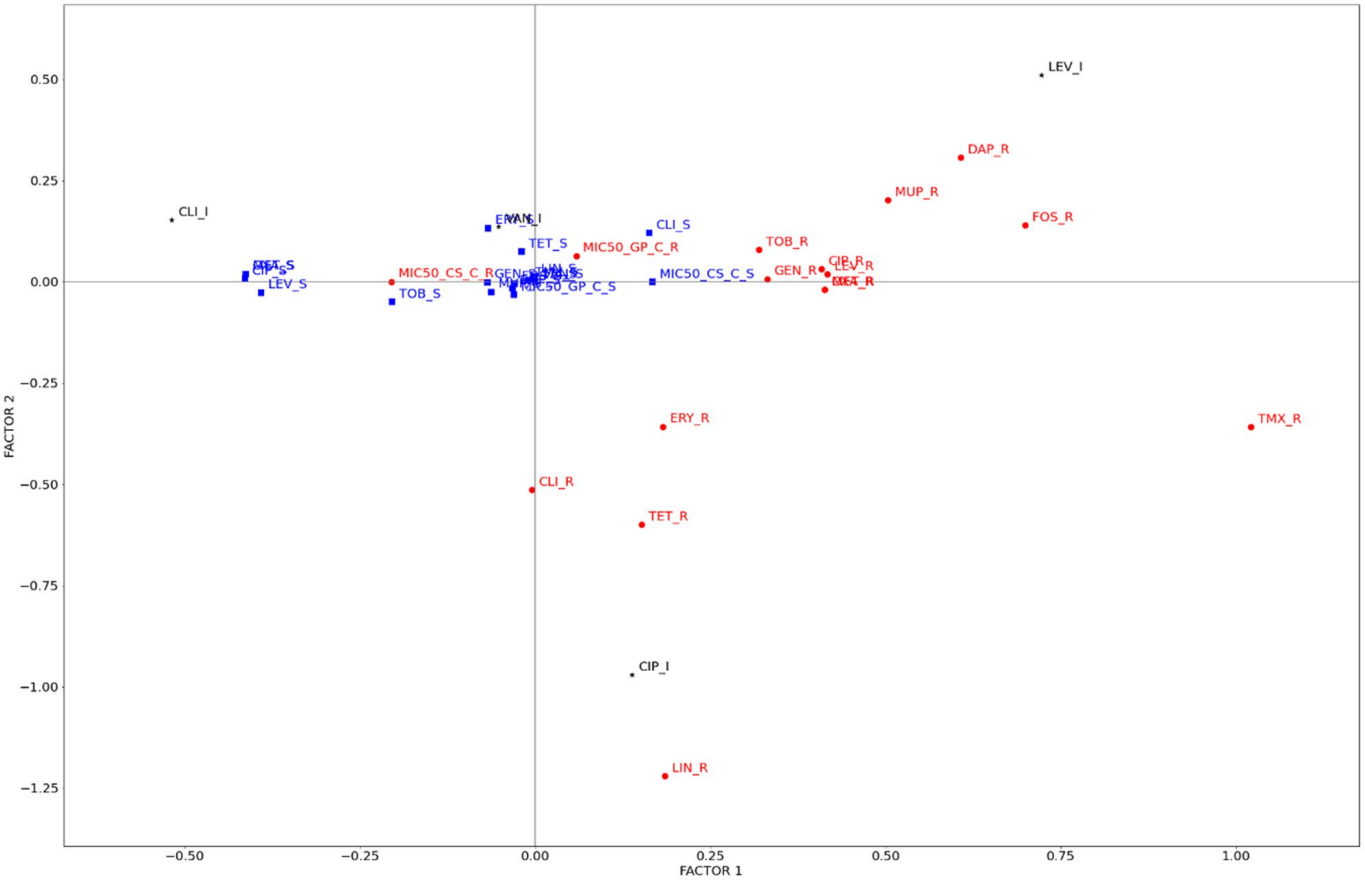
MCA results for categorical values (resistance, sensitiveness or intermediate) as points. Distribution of the different groups of isolates divided based on their resistance or sensitivity to the different clinical antibiotics and extracts tested. R (red text and red circles), I (black text and black * symbols) and S (blue text and blue squares) indicate resistant, intermediate and sensitive, respectively. Two magnified inserts and connections between antibiotics have been included for clarification in Supplementary Fig. S5.

The second approach was based on a direct representation of categorical values (resistant or not to a single antibiotic/extract) as points. Intermediate sensitivity was also included for vancomycin (VAN), clindamycin (CLI), levofloxacin (LEV) and ciprofloxacin (CIP). No data for VAN-resistant or teicoplanin (TEI)-resistant isolates were included, as none of the isolates presented these characteristics. For this analysis, the susceptibility to CS and GP extracts was determined by comparing the individual MIC_50_ value for each isolate with the mean MIC_50_ value for the whole 100 isolates population (65.34 and 62.96 µg/mL for CS and GP respectively). If the individual MIC_50_ was higher than the mean value for all the isolates, that single isolate was considered as resistant. Otherwise if the value was lower, the isolate was classified as sensitive. In this analysis, the first dimension accounted for 44.17% of the variance, and the second accounted for 9.85%, accounting for a total of 54.02% of the variance. A joint plot of category points was obtained, and is shown in Fig. 6. Category quantification plots constitute an alternative method of displaying discrimination of variables that can identify categorical relationships.

The results shown in Fig. 6 clearly group most of the points close to the horizontal axis (Factor 2 = 0), suggesting that this second dimension has little influence on the point distribution, and shows a greater influence of the F1 factor on this distribution. Additionally, most of the points are close to the axis intercept (0:0 point), indicating that they are not influenced by any of the values of either dimension. After this preliminary conclusion, some interesting results could be extracted from this analysis. Most of the points showing resistance to an antibiotic are on the right side of the plot (positive F1 values), whereas the majority of points associated with antibiotic sensitivity are on the left side. There are only two exceptions to this general conclusion, clindamycin (CLI), whose resistant point (CLI-R) is on the left side of the graph, and CS extract, whose points are also inverted. This last result indicates that the sensitivity of the isolates to the CS extract is related to their resistance to almost all of the antibiotics, confirming the results obtained in Fig. 4. In other words, the CS extract is especially active against antibiotic-resistant isolates.

Another relevant result was related to the mechanism of action of the different antibiotics. The antibiotics and extracts were divided into five categories according to their putative mechanism: protein synthesis inhibitors (GEN, TOB, ERI, CLI, TET, LIN and MUP), cell division inhibitors (CIP and LEV), plasmatic membrane disruptors (DAP) and cell wall disruptors (MET, OXA, FOS, VAN and TEI) and other mechanisms (TMX and the CS and GP extracts). Supplementary Fig. S5 online shows a complementary version of the MCA plot showing these mechanisms. Surprisingly, antibiotics presenting plasmatic membrane and cell wall disruption mechanisms are aligned with the F1 axis, with F2 values close to 0. This profile is similar for both the CS and GP extracts, suggesting that their putative mechanisms may be related to membrane or cell wall alterations. On the other hand, antibiotics that inhibit both protein synthesis and cell division showed a wider distribution, hampering our ability to draw any conclusions in addition to their relationship with both the F1 and F2 dimensions.

## Discussion

In the present work, natural pure compounds and extracts, previously selected for their potential antimicrobial capacity, have been challenged against different microorganisms with clinical relevance to study their putative use as new antimicrobial agents. A two-step screening design was used to select the best candidates for further studies. This first screening utilized 105 clinical isolates of 11 different strains (3 Gram-negative and 8 Gram-positive bacteria), and it revealed that three pure compounds and two extracts were active against *S. aureus* and *S. maltophilia.* As the number of available isolates of *S. maltophilia* was very low in our geographical area, the study was therefore focused on *S. aureus*. When the results for this microorganism were analyzed, only three pure compounds (P, Q3G and GA) and two extracts (CS and GP) presented positive results, especially the compound P and the CS and GP extracts. In addition, P was also the main compound in both extracts. These compounds and extracts have been studied previously because of their antimicrobial activity on *S. aureus*, confirming their relevance for future developments^3^.

In general, it was observed that the CS extract was more effective against antibiotic-resistant bacteria than against antibiotic-sensitive bacteria, while the GP extract was more effective against bacteria sensitive to common clinical antibiotics. This tendency was evidenced when the CS extract was used against bacteria resistant to beta-lactam antibiotics, with statistical significance (*p* < 0.05, *). Significant differences were also observed in GP extract against bacteria sensitive to quinolones and oxacillin (*p* < 0.05, *). These results were reinforced by using GLMR and MCA analysis. These results were also consistent with those obtained by Atef M. et al., in which differential antimicrobial activity of plant extracts against several strains of *P. aeruginosa* was observed and correlated with different antibiotic resistance profiles^14^.

In the present study, the average MIC_50_ value of the CS extract against clinical isolates of MSSA was 80.67 μg/mL, whereas it was 51.21 μg/mL against MRSA isolates. The antimicrobial capacity of different *C. salviifolius* extracts has been reported previously by our group and others, resulting in similar MIC_50_ values: 11 μg/mL^12^ and between 45 and 50 μg/mL^9^, both against *S. aureus* CECT 59 (MSSA). Regarding MRSA, a previous study quantified the MIC_50_ of a CS extract against a clinically isolated MRSA strain from Libya at 25 mg/mL^15^. This MIC_50_ value was much higher than that obtained from our CS extract. This difference may be due to the different methods utilized to obtain the CS extract. The extract used by Abdurrezagh E. et al. was obtained by conventional extraction using a mixture of methanol and water (8:2, v/v) filtered and dried without any further purification step and without providing any other analytical data. Our extract was obtained without using alcohol and was subjected to a final column fractionation step using FPX66 resin to obtain the polyphenol-enriched (60% w/w) extract fraction. The different antimicrobial capacity might also be due to the different *C. salviifolius* raw material utilized to produce the extract, since the level of polyphenols in the plants can vary based on the environmental conditions, stresses or even the time of year in which collection occurred^16^.

The present study determined that the average MIC_50_ value against MSSA of the GP extract was 51.67 µg/mL, and its value against MRSA was 72.89 μg/mL. Other studies determined the antimicrobial effect of dried pomegranate peel extracts against MSSA (ATCC 11632) and MRSA (ATCC 33591), presenting MIC_50_ values between 100 and 250 μg/mL for both MSSA and MRSA^17^. According to the manufacturer’s information, the GP extract was also obtained after a purification process using an affinity resin, similar to that utilized to obtain CS extract. This process ensures a high polyphenolic content that can be, as occurred with the CS extract, the explanation for the better results of this study when compared with those in the literature.

Another aspect that deserves discussion is the fact that the two polyphenolic extracts utilized in the present study showed very different activities depending on the bacteria tested. This was very likely due to their different polyphenolic composition. The characterization of the extracts performed by HPLC–MS determined that the most abundant quantitative compounds in both extracts were hydrolyzable tannins, which showed 32.800% and 30.460% relative contents in the CS and GP extracts, respectively. Therefore, the differences in activity should depend on the less abundant compounds. The antimicrobial activity of punicalagin alone was tested against 7 different *S. aureus* isolates (ATCC 29213, ATCC 25923 and 5 laboratory isolates from food) and showed a MIC value of 250 μg/mL^18^. While the GP extract only contained hydrolyzable tannins (ellagitannins and gallotannins) and one flavonol at very low concentration, the CS extract contained more flavonoids (flavones, flavonols and flavanols), phenolic acids and a coumarin. Previous studies related the structure of certain polyphenolic compounds to their differential activity against sensitive and resistant antibiotic bacteria. Regarding MRSA, polyphenols presenting certain chemical groups, such as COOH and OH groups in the *ortho* and *para* positions, or an O–CH_3_ group in the *meta* position of the benzene ring, seemed to have increased specific anti-MRSA activity^19^; the flavonoids presented in the CS extract but not in the PG extract present these characteristics. The higher activity of the CS extract against antibiotic-resistant bacteria could also be explained by the synergistic activity of flavonoids and hydrolyzable tannins at certain concentrations as previously described by our group^12^. The presence of punicalin and punigluconin, hydrolyzable tannins not present in the GP extract, could also explain part of the increased activity of the CS extract, although it is unlikely since these were present at low concentrations.

Based on the results obtained by using GLMR and MCA statistical approaches, we can conclude that the bacterial susceptibility to the studied extracts is related to the antibiotic resistance profile of the clinical isolates of *S. aureus*. Specifically, it was observed that bacterial isolate resistance to beta-lactam antibiotics and quinolones was directly related to their susceptibility to the action of the CS extract. In contrast, PG extract was more effective on bacteria sensitive to quinolones and oxacillin. On the other hand, MCA based on categorical values suggested a mechanism related to plasmatic membrane or cell wall disruption. This result is consistent with that previously reported which postulated these mechanisms as the main molecular target for polyphenols such as hydrolyzable tannins^3^. Nevertheless, further mechanistic studies must be developed to confirm this hypothesis.

In conclusion, CS and GP extracts demonstrated the highest antimicrobial activity, among all the natural antimicrobial agents used, against a panel of eleven different microbial species with clinical relevance. Moreover, CS extract exhibited higher antimicrobial capacity against resistant *S. aureus* clinical isolates, whereas PG was more effective against sensitive ones. The use of generalized linear model regression and multiple correspondence statistical analysis revealed that extracts antimicrobial capacity depended on the clinical antibiotic resistance profile of each bacterial isolate. The CS extract, which contained hydrolyzable tannins and flavonoids, was more effective against beta-lactam-resistant bacteria, while the GP extract, which contained mostly hydrolyzable tannins, was more effective against quinolone and oxacillin-sensitive bacteria. We postulate that the superior activity of the CS extract against antibiotic-resistant bacteria may be related either to the presence of flavonoids or to a synergistic interaction between hydrolyzable tannins and flavonoids. A link between bacterial susceptibility to GP and CS extracts with the resistance profile based on cell wall disruption mechanism is also proposed. These observations open further possibilities for future studies focused on the relationship between bacterial resistance to certain classes of antibiotics and natural molecules with an enhanced antimicrobial activity. This approach may enable to develop personalized combined antibiotic therapies for treating resistant infections with greater efficacy and less dependence on chemically synthesized molecules.

## Materials and methods

### Ethical statement

All the procedures and methods used in this study were performed in accordance with the relevant guidelines and regulations and were previously approved by the Universidad Miguel Hernández Ethics and integrity for Research Committee with the reference UMH.IBM.VMM.05.15.

This committee waived the need for an additional informed consent for this study because all the samples were obtained during the usual clinical practice at the University General Hospital of Alicante and were covered by its corresponding informed consent. These consents remain in the custody of the hospital and were incorporated to the clinical history of each patient. Those informed consents explicitly include future uses for research purposes always maintaining patient’s anonymity.

### Botanical extract and pure compound selection and procurement

The following pure compounds were purchased from Sigma-Aldrich (Misuri, USA): gallic acid, punicalagin, quercetin-3-glucuronide, myricetin, naringenin and ellagic acid *Punica granatum* and *Citrus paradisi* fruit extracts were obtained from the companies Monteloeder S.L. and Nutracitrus S.L., respectively. *C. salviifolius* extract was produced in the laboratory by aqueous extraction of leaves followed by a column chromatography purification process using AmberLite FPX66 resin (DuPont, Delaware, USA) and characterized as described in^8,9^. *C. salviifolius* specimens were grown at Universidad Miguel Hernández facilities in Elche, Alicante (Spain) and harvested after being identified by the authors. Representative samples were deposited and preserved in their sample collection as CS190723-EBC.

### Antimicrobial assays

For the disk diffusion assay, a known amount of compound or extract (40 µg) diluted in 5 µL of distilled water was applied on small discs of paper (5 mm of diameter) placed in a petri dish with microorganisms previously sown extensively over a semisolid Mueller–Hinton agar (Merck Millipore, Massachusetts, USA). Positive (discs with antibiotic) and negative (disc with phosphate-buffered saline adjusted to pH 7.4) were included in all the dishes. A halo of inhibition was generated around the discs impregnated with antimicrobial activity. Every test was performed in duplicate. These antimicrobial assays were performed on isolates of the following microbial species: *Staphylococcus aureus*, *Enterococcus faecalis*, *Enterococcus faecium*, *Escherichia coli*, *Klebsiella pneumoniae*, *Enterobacter *spp., *Serratia marcescens*, *Salmonella *spp., *Pseudomonas aeruginosa*, *Acinetobacter baumannii* and *Stenotrophomonas maltophilia*. These species were chosen for their great importance in the health field due to their clinical impact and development of drug resistance. The microorganisms were obtained from clinical samples of patients at the General University Hospital of Alicante.

The antibiotic susceptibility of each single isolate was tested following EUCAST procedures and CLSI standards. All the isolates were classified as resistant (R), sensitive (S) or intermediate (I) according to the EUCAST classification and listed on Supplementary Table S3.

Plate microdilution assays were performed in 96-well plates. Microcultures of different isolates of *S. aureus* with 10 different concentrations ranging between 2 mg/mL and 0.0004 mg/mL of antimicrobial extract were carried out in each plate. Positive (ciprofloxacin) and negative (extracts vehicle and non-inoculated sample) controls were included in all the plates. Each plate was performed in duplicate. The culture medium used was Mueller–Hinton broth (Merck Millipore, Massachusetts, USA). After 24 h of incubation at 37 °C, the wells with medium and bacteria were stained with iodonitrotetrazolium chloride (Sigma-Aldrich, Misuri, USA) to stain viable bacteria red. After 30 min of plate incubation at 37 °C, the absorbance at 570 nm was measured using a spectrophotometer (BioTek Synergy HTX, Vermont, USA) to determine the microbial proliferation in each well. The absorbance values obtained were proportional to the number of viable bacteria stained in each well. The data obtained were analyzed using GraphPad Prism 6 software.

### Total phenolic content determination

The total phenolic content of the extracts was measured by using the gallic acid equivalence (GAE) method in 96-well plates^20^. First, 10 μL of each sample was mixed with 50 μL of Folin–Ciocalteu’s phenol reagent. After 1 min, 100 µL of Na_2_CO_3_ solution (20%, w/v) and 840 µL of distilled water were added to the mix. The reaction was kept in dark for 30 min. Plate absorbance was measured at 700 nm using a spectrophotometer (BioTek Synergy HTX, Vermont, USA). A standard curve of gallic acid (Sigma-Aldrich, Misuri, USA) was previously prepared using solutions of a known concentration in water. The absorbance data obtained were analyzed using GraphPad Prism 6 software. Results were expressed in terms of gallic acid equivalents (g GAE/100 g of dry matter of plant).

### High-performance liquid chromatography analysis

The molecular composition of the extracts selected in the first screening was analyzed by high-performance liquid chromatography coupled to mass spectrometry (HPLC–MS) using an Agilent LC 1100 series (Agilent Technologies, Inc., Palo Alto, CA, USA) coupled to an Esquire 3000+ (Bruker Daltonics, GmbH, Germany) mass spectrometer as described previously^12^. Briefly, HPLC instrument was equipped with a pump, autosampler, UV–vis diode array detector, and column oven. The HPLC instrument was controlled by Chemstation software. The mass spectrometer instrument was equipped with an electrospray ionization (ESI) source and ion-trap mass analyzer. Mass spectrometer was operated by Esquire Control and DataAnalysis 3.4 software. The chromatographic column used was an Agilent Poroshell 120 RP—C18 column (4.6 × 150 mm, 2.7 µm).

The method used for sample separation consisted of a linear gradient of 1% formic acid (A) and acetonitrile (B). Gradient started at 5% of B, increasing to 25% of B at 30 min, to 45% of B at 45 min, then 5% of B at 51 min and for an additional 5 min for column re-equilibration purposes. The flow rate was constant at 0.5 mL/min. The diode-array detector was set at 280, 320 and 340 nm. The ESI ionization source was operated in negative mode to generate [M−H]^−^ ions. ESI conditions were set as follows: desolvation temperature, 360 °C; vaporizer temperature, 400 °C; dry gas (nitrogen), 12 L/min; nebulizer, 70 psi. Full scan mode from 50 to 1400 m/z with an ion trap collection time set at 200 ms was used for data acquisition.

Identification of the main compounds was performed by HPLC–DAD analysis using a home-made library of phenolic compounds and comparing the retention times, UV spectra and MS/MS data of the peaks in the samples with those of authentic standards or data reported in the literature^7^. The interpretation of the spectra and identification of the main compounds was carried out using DataAnalysis 3.4 software (Bruker Daltonics, GmbH, Germany).

Quercetin was used as the representative flavonoid to quantify this polyphenolic compound group. Punicalagin was used as the representative hydrolyzable tannin to quantify this polyphenolic compound group. Both punicalagin and quercetin used for the standard curves in the quantification of polyphenolic compounds of the extracts were purchased from Merck (Germany). The software ChemStation for LC 3D (Agilent Technologies Life Sciences and Chemical Analysis, Waldbronn, Germany) was used for quantitation purposes. The linearity range of the responses was determined on eight concentration levels (ranging from 0.25 to 0.25 mg/mL) with three injections for each level. Calibration graphs for the quantitative evaluation of the compounds were performed by means of a six-point regression curve (r^2^ > 0.996)^7^.

### Statistical analysis

The minimum concentration that inhibits the bacterial growth by 50% (MIC_50_) for each isolate and significant differences between treatments and data sets were calculated using GraphPad Prism 6 by processing the data obtained in the microdilution antimicrobial assays. The data gathered in the assays were analyzed using a nonlinear fit with least squares (log inhibitor vs normalized response with variable slope, equation: Y = 100/(1 + 10^((LogIC_50_ − X) × HillSlope))) to calculate MIC_50_ values. Final graphs were generated using Microsoft Excel 2016. Generalized linear model regression (GLMR) and multiple correspondence analysis (MCA) were performed using Microsoft Excel and Google Colab with Jupyter Notebooks, libraries mca-1.0.3, Pandas v0.25.3, and Matplotlib Python v3.2.0. On the one hand, the term GLMR usually refers to conventional linear regression models for a continuous response variable given continuous and/or categorical predictors. In this case, the response variable is assumed to follow a normal family distribution. On the other hand, MCA takes multiple categorical variables and seeks to identify associations between levels of those variables. It can be thought of as analogous to principal component analysis for quantitative variables. Similar to other multivariate methods, it is a dimension reducing method resenting the data as points in a 2-dimensional space.

## Supplementary Information

Supplementary Information.

## Data Availability

Most data generated or analyzed during this study are included in this published article (and its Supplementary Information files). The datasets generated during and/or analyzed during the current study that are not present in the article or its Supplementary Information files are available upon justified request to the corresponding author.
