# Author Correction: The antimicrobial capacity of *Cistus salviifolius* and *Punica granatum* plant extracts against clinical pathogens is related to their polyphenolic composition

**DOI:** 10.1038/s41598-023-39602-8

**Published:** 2023-08-10

**Authors:** Francisco Javier Álvarez‑Martínez, Juan Carlos Rodríguez, Fernando Borrás‑Rocher, Enrique Barrajón‑Catalán, Vicente Micol

**Affiliations:** 1 Instituto de Biología Molecular y Celular (IBMC) and Instituto de Investigación, Desarrollo e Innovación en Biotecnología Sanitaria de Elche (IDiBE), Universidad Miguel Hernández (UMH), 03202 Elche, Spain; 2 Microbiology Section, University General Hospital of Alicante, Alicante Institute for Health and Biomedical Research (ISABIAL Foundation, Alicante, Spain; 3 Statistics and Operative Research Department, Miguel Hernández University (UMH), Avda. Universidad s/n, 03202 Elche, Spain; 4 CIBER, Fisiopatología de la Obesidad y la Nutrición, CIBERobn, Instituto de Salud Carlos III (CB12/03/30038), Madrid, Spain

Correction to: *Scientific Reports* https://doi.org/10.1038/s41598-020-80003-y, published online 12 January 2021

The original version of this Article contained errors in the values of total phenolic content.

As a result, in the Results section, under the subheading ‘Phenolic content and HPLC–MS molecular characterization of the extracts’,

“The CS extract showed a total phenolic content of 60.1 ± 5.5 mg GAE/g extract. In contrast, the GP extract showed a lower total phenolic content with 35.9 ± 2.5 mg GAE/g extract.”

now reads:

“The CS extract showed a total phenolic content of 60.1 ± 5.5 g GAE/100 g extract. In contrast, the GP extract showed a lower total phenolic content with 35.9 ± 2.5 g GAE/100 g extract.”

The original Article has been corrected.

