# Supplementary material for: The antimicrobial capacity of Cistus salviifolius and Punica granatum plant extracts against clinical pathogens is related to their polyphenolic composition
Source: Sci Rep. 2021 Jan 12;11:588. doi: 10.1038/s41598-020-80003-y (PMC7803989; doi:10.1038/s41598-020-80003-y)
Supplement: Supplementary file 1 — Supplementary Information. [file 41598_2020_80003_MOESM1_ESM.pdf]

The antimicrobial capacity of polyphenolic extracts against resistant clinical pathogens is related to their flavonoid and ellagitannin composition.

Francisco Javier Álvarez-Martínez [1], Juan Carlos Rodríguez [2], Fernando Borrás-Rocher [3], Enrique Barrajón-Catalán [1,*,#] and Vicente Micol [1,4,#].

[1] Instituto de Biología Molecular y Celular (IBMC) and Instituto de Investigación, Desarrollo e Innovación en Biotecnología Sanitaria de Elche (IDiBE), Universidad Miguel Hernández; 03202, Elche, Spain.
[2] Microbiology Section, University General Hospital of Alicante, Alicante Institute for Health and Biomedical Research (ISABIAL Foundation).
[3] Statistics and Operative Research Department, Miguel Hernández University (UMH), Avda. Universidad s/n, 03202, Elche, Spain.
[4] CIBER, Fisiopatología de la Obesidad y la Nutrición, CIBERobn, Instituto de Salud Carlos III (CB12/03/30038).
# These authors shared author co-seniorship.

* Corresponding author: Enrique Barrajón-Catalán, Instituto de Biología Molecular y Celular (IBMC) and Instituto de Investigación, Desarrollo e Innovación en Biotecnología Sanitaria de Elche (IDiBE), Universidad Miguel Hernández; 03202, Elche, Spain.

Tel.: +0034 965222586

E-mail adress: e.barrajon@umh.es (E. Barrajón-Catalán)

Supplementary Material

Families of polyphenols

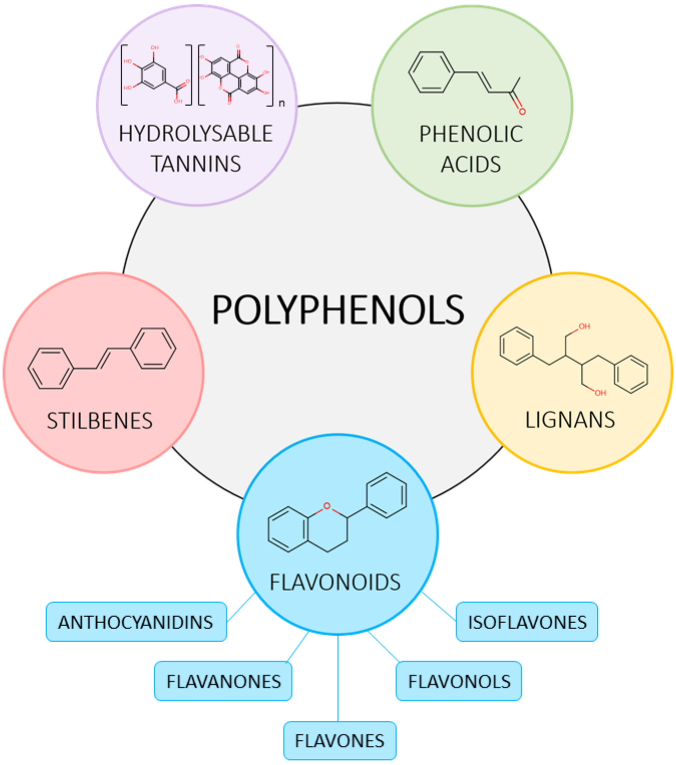

Supplementary Figure S1*. Families of polyphenols with their core chemical structures.*

Standard curves

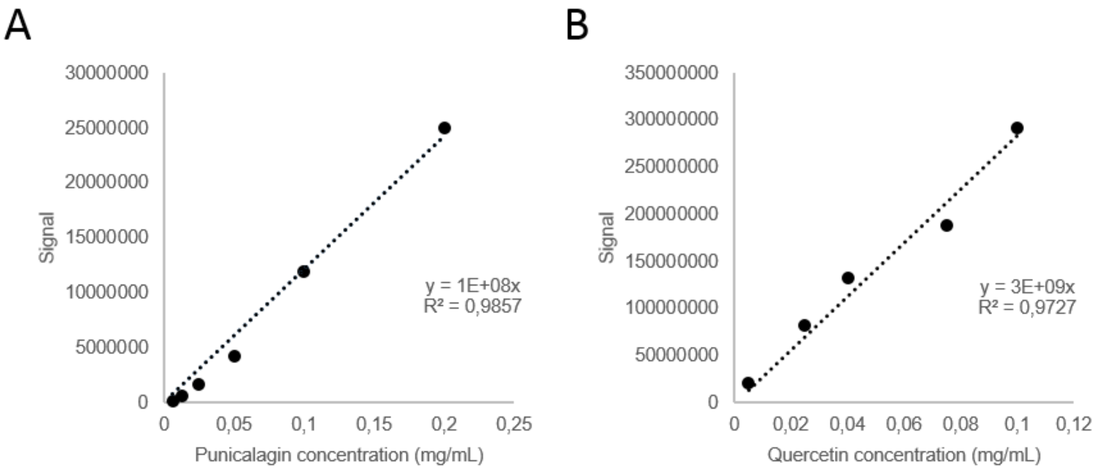

Supplementary Figure S2. *Standard curves for punicalagin (A) and quercetin (B).*

Main compounds in CS and GP extracts

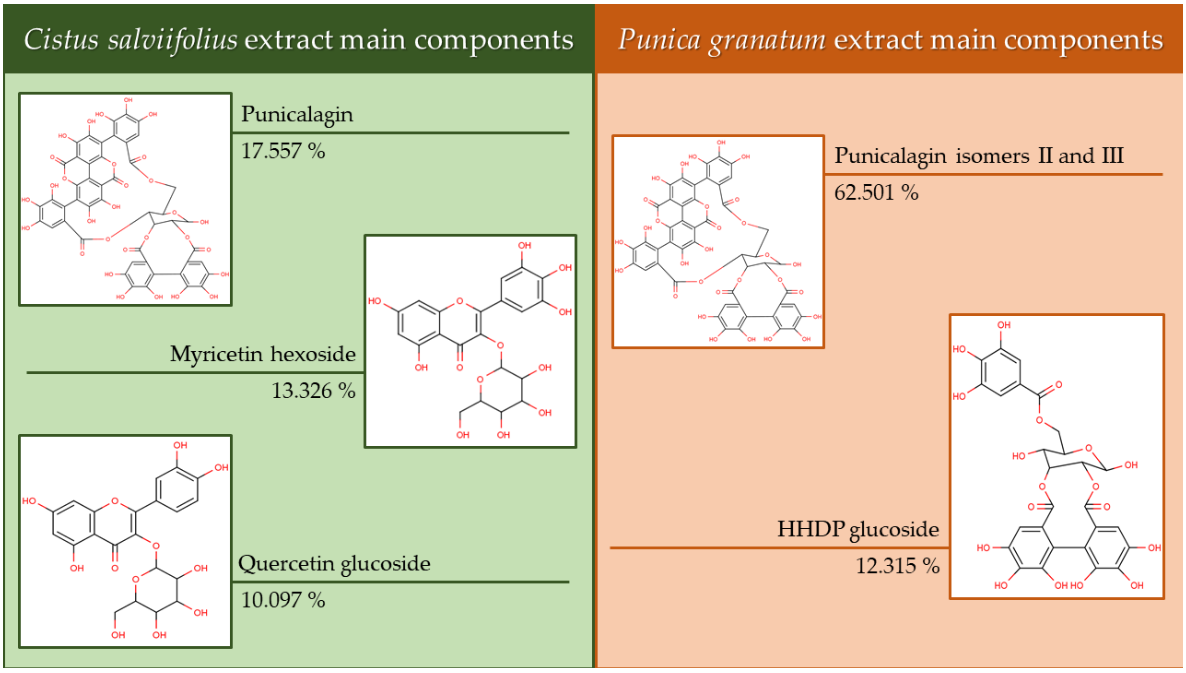

Supplementary Figure S3. *Compounds with higher relative abundance identified on CS (green, left) and GP (orange, right) extracts.*

Distribution of the MIC_{50} values by extract and antibiotic resistance profile

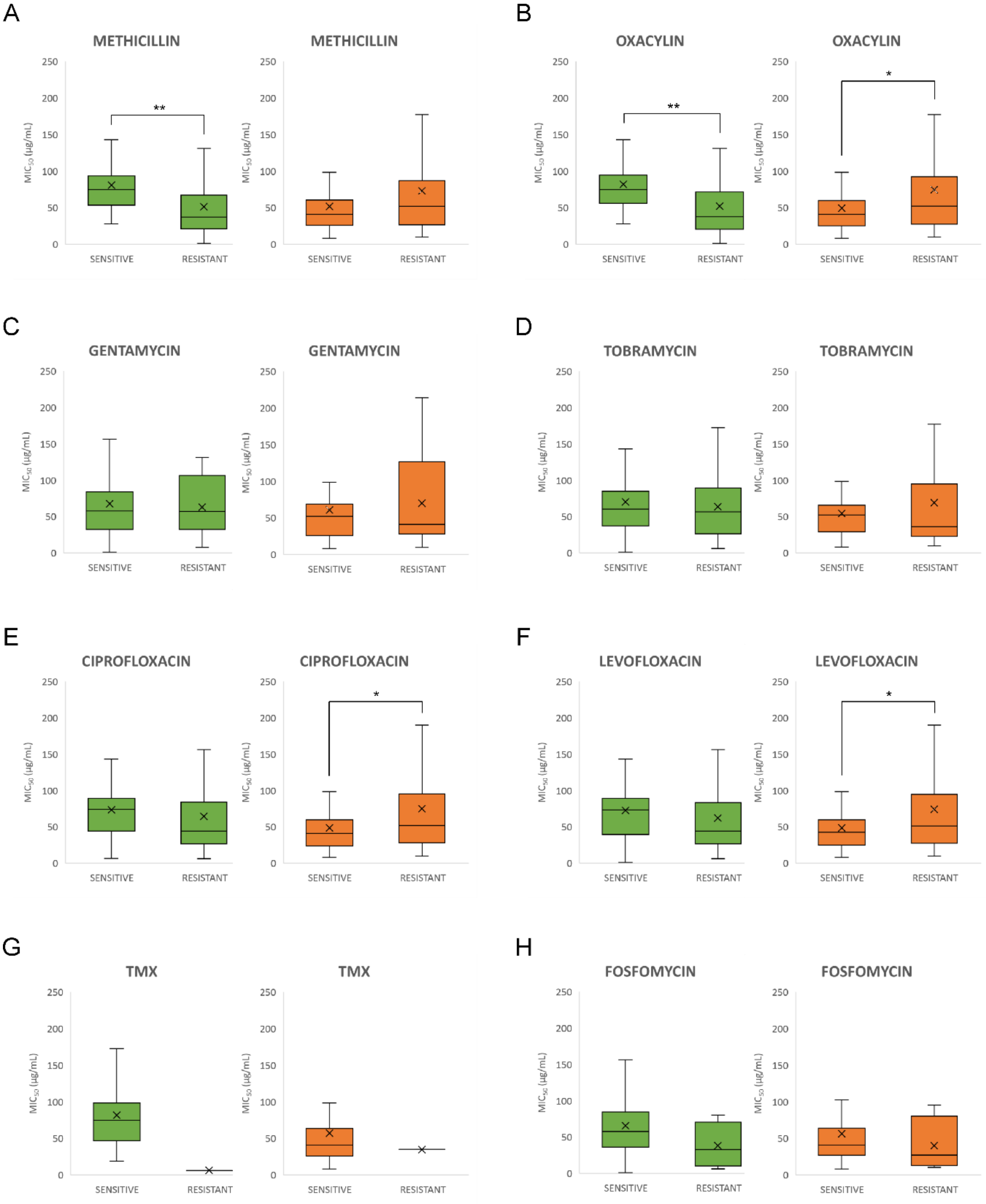
A
B
C
D
E
F
G
H
METHICILLIN
OXACYLIN
GENTAMYCIN
TOBRAMYCIN
CIPROFLOXACIN
LEVOFLOXACIN
TMX
FOSFOMYCIN
MIC50 (µg/mL)
SENSITIVE
RESISTANT
**
*
0
50
100
150
200
250

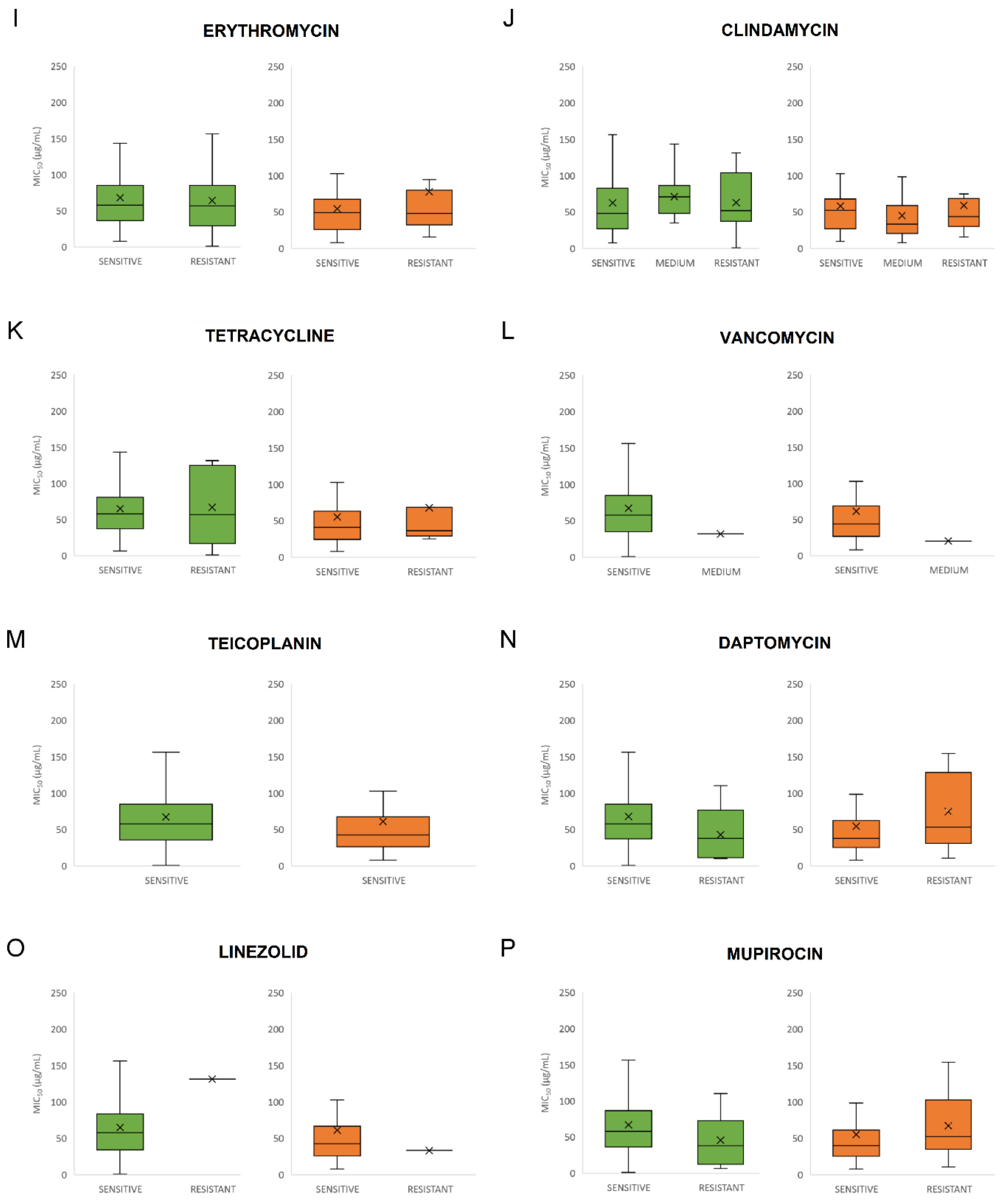

Supplementary Figure S4. *Distribution of the MIC$_{50}$ values (µg/mL) of every S. aureus isolate grouped by single antibiotic resistances and extract used (green for CS and orange for GP).*

MCA results

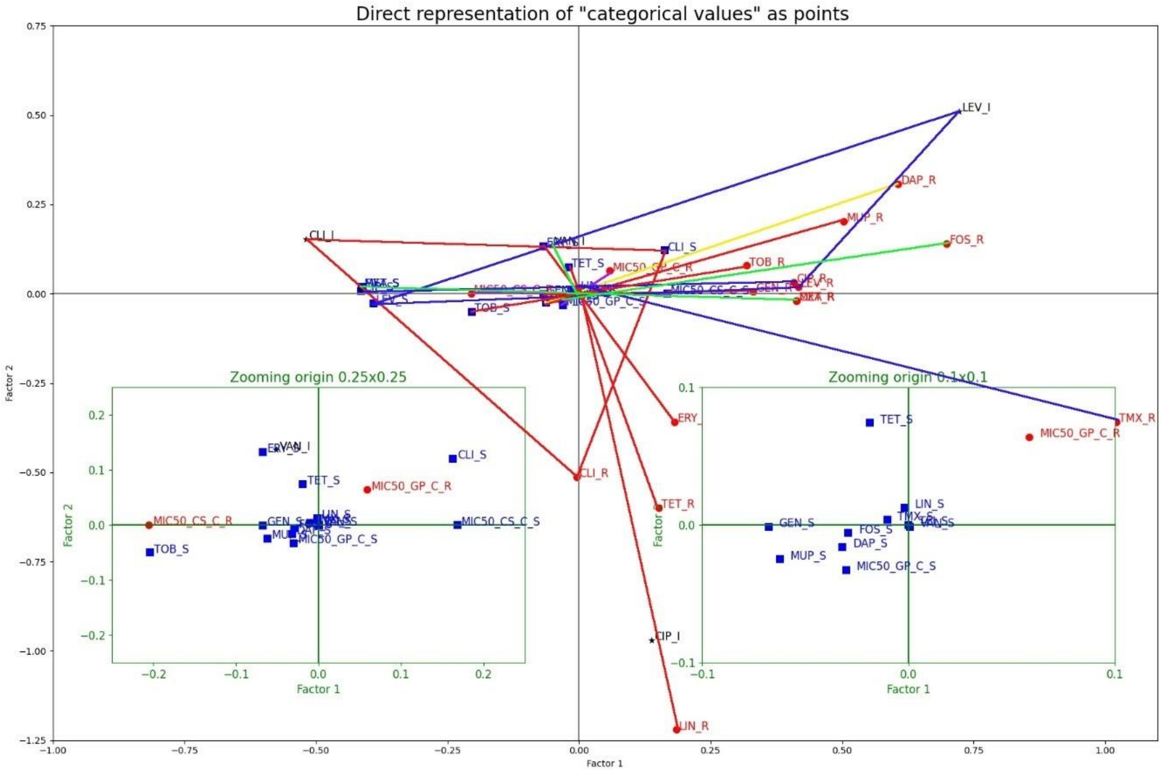

Supplementary Figure S5. *MCA results for "categorical values" as points. Distribution of the different groups of isolates divided based on their resistance or sensitivity to the different clinical antibiotics and extracts tested. MET: methicillin, OXA: oxacillin, GEN: gentamicin, TOB: tobramycin, CIP: ciprofloxacin, LEV: levofloxacin, TMX: trimetrexate, FOS: fosfomycin, ERY: erythromycin, CLI: clindamycin, TET: tetracycline, VAN: vancomycin, TEI: teicoplanin, DAP: daptomycin, LIN: linezolid, MUP: mupirocin, CS: Cistus salviifolius extract, GP: Punica granatum extract. (R, red text and red circles), (I, black text and black * symbols) and (S, blue text and blue squares) mean Resistant, Intermediate and Sensitive, respectively. Two zoomings inserts have been included to a better understanding. Points related to the same antibiotic are connected by a line whose color is related to the mechanism of action: red for protein synthesis inhibitors, blue for cell division inhibitors, yellow for plasmatic membrane disruptors, green for cell wall disruptors and black for others mechanisms.*

MIC_{50} of extracts against MRSA

Species	Isolate	CS MIC_{50}	GP MIC_{50}
S. aureus	1	38.585	61.165
S. aureus	2	21.115	68.210
S. aureus	3	30.450	26.380
S. aureus	4	28.174	44.460
S. aureus	5	32.005	20.121
S. aureus	6	38.115	74.680
S. aureus	7	20.425	58.950
S. aureus	8	16.640	25.045
S. aureus	9	26.735	82.410

S. aureus	10	44.235	40.020
S. aureus	11	220.220	84.520
S. aureus	12	37.325	53.165
S. aureus	13	14.078	97.185
S. aureus	14	47.080	64.013
S. aureus	15	63.060	62.570
S. aureus	16	6.382	36.390
S. aureus	17	25.205	52.660
S. aureus	18	109.000	14.845
S. aureus	19	7.977	272.600
S. aureus	20	37.000	189.900
S. aureus	21	31.015	37.080
S. aureus	22	80.390	26.830
S. aureus	23	18.140	70.540
S. aureus	24	30.120	24.860
S. aureus	25	41.900	19.770
S. aureus	26	0.944	51.890
S. aureus	27	23.535	95.330
S. aureus	28	18.780	281.400
S. aureus	29	73.220	241.100
S. aureus	30	58.920	35.190
S. aureus	31	29.145	71.115
S. aureus	32	172.400	94.540
S. aureus	33	40.835	26.755
S. aureus	34	7.488	9.764
S. aureus	35	80.110	10.430
S. aureus	36	156.000	177.100
S. aureus	37	27.790	30.485
S. aureus	38	64.960	52.215
S. aureus	39	124.900	28.865
S. aureus	40	74.250	150.200
S. aureus	41	38.050	19.235
S. aureus	42	131.000	15.250
S. aureus	43	112.225	40.200
S. aureus	44	57.040	214.100
S. aureus	45	37.920	10.660

S. aureus	46	110.105	154.185
S. aureus	47	12.400	102.675
S. aureus	48	26.800	37.730
S. aureus	49	10.052	51.175
S. aureus	50	6.162	34.665

***Supplementary Table S1**. MIC_{50} (µg/mL) values of the CS and GP extracts against 50 MRSA clinical isolates.*

MIC_{50} of extracts against MSSA

Species	Isolate	CS MIC_{50}	GP MIC_{50}
S. aureus	51	89.560	54.760
S. aureus	52	83.920	66.710
S. aureus	53	>252.300	71.290
S. aureus	54	>252.300	54.340
S. aureus	55	252.300	>174.800
S. aureus	56	96.930	31.240
S. aureus	57	92.260	66.500
S. aureus	58	105.500	21.810
S. aureus	59	57.520	30.810
S. aureus	60	27.780	25.180
S. aureus	61	74.710	60.710
S. aureus	62	74.510	52.490
S. aureus	63	31.000	157.500
S. aureus	64	70.720	8.152
S. aureus	65	88.090	57.090
S. aureus	66	>252.300	98.470
S. aureus	67	78.460	59.510
S. aureus	68	39.050	>174.800
S. aureus	69	142.900	20.280
S. aureus	70	56.540	>174.800
S. aureus	71	57.490	40.970
S. aureus	72	105.300	34.130
S. aureus	73	34.950	32.220
S. aureus	74	74.920	28.390
S. aureus	75	214.200	>174.800
S. aureus	76	72.320	22.740
S. aureus	77	55.050	20.510
S. aureus	78	41.180	>174.800

S. aureus	79	78.530	14.380
S. aureus	80	>252.300	18.360
S. aureus	81	78.500	48.970
S. aureus	82	73.900	43.800
S. aureus	83	57.740	34.400
S. aureus	84	76.330	30.110
S. aureus	85	74.050	23.510
S. aureus	86	37.140	56.990
S. aureus	87	79.130	133.700
S. aureus	88	123.500	19.280
S. aureus	89	46.690	>174.800
S. aureus	90	113.500	174.800
S. aureus	91	57.760	163.400
S. aureus	92	102.900	23.570
S. aureus	93	49.280	54.790
S. aureus	94	37.760	68.700
S. aureus	95	43.040	53.590
S. aureus	96	128.700	27.590
S. aureus	97	83.200	33.160
S. aureus	98	37.170	40.880
S. aureus	99	84.960	60.490
S. aureus	100	131.200	33.130

Supplementary Table S2*. MIC$_{50}$ (µg/mL) values of the CS and GP extracts against 50 MSSA clinical isolates.*

S. aureus isolates antibiotic resistance profiles

**Isolate**	**MET**	**OXA**	**GEN**	**TOB**	**CIP**	**LEV**	**TMX**	**FOS**	**ERY**	**CLI**	**TET**	**VAN**	**TEI**	**DAP**	**LIN**	**MUP**
1	R	R	S	S	S	S	S	S	S	S	S	S	S	S	S	S
2	R	R	S	S	S	S	S	S	S	S	S	S	S	S	S	S
3	R	R	S	S	S	S	S	S	S	S	S	S	S	S	S	S
4	R	R	S	S	S	S	S	S	S	S	S	S	S	S	S	S
5	R	R	S	S	S	S	S	S	S	S	S	I	S	S	S	S
6	R	R	S	S	S	S	S	S	R	R	S	S	S	S	S	S
7	R	R	S	S	R	R	S	S	S	S	S	S	S	S	S	S
8	R	R	S	S	I	S	S	S	S	S	R	S	S	S	S	S
9	R	R	S	S	R	R	S	S	S	S	S	S	S	S	S	S
10	R	R	S	S	R	R	S	S	S	S	S	S	S	S	S	S
11	R	R	S	S	R	R	S	S	S	S	S	S	S	S	S	S

12	R	R	S	S	R	R	S	S	S	S	S	S	S	S	S	S
13	R	R	S	S	R	R	S	S	S	S	S	S	S	S	S	S
14	R	R	S	S	R	R	S	S	S	S	S	S	S	S	S	S
15	R	R	S	S	R	R	S	S	R	S	S	S	S	S	S	S
16	R	R	S	S	S	S	S	S	R	R	R	S	S	S	S	S
17	R	R	S	R	R	R	S	S	S	S	S	S	S	S	S	S
18	R	R	S	R	R	R	S	S	S	S	S	S	S	S	S	S
19	R	R	S	R	R	R	S	S	S	S	S	S	S	S	S	S
20	R	R	S	S	R	R	S	S	S	S	R	S	S	S	S	S
21	R	R	S	R	R	R	S	S	S	S	S	S	S	S	S	S
22	R	R	S	R	R	R	S	S	S	S	S	S	S	S	S	S
23	R	R	S	R	R	R	S	S	S	S	S	S	S	S	S	S
24	R	R	S	S	R	R	S	S	R	S	S	S	S	S	S	S
25	R	R	S	S	R	R	S	R	S	S	S	S	S	S	S	S
26	R	R	S	S	I	S	S	S	R	R	R	S	S	S	S	S
27	R	R	S	R	R	I	S	R	S	S	S	S	S	S	S	S
28	R	R	S	R	R	R	S	S	R	S	S	S	S	S	S	S
29	R	R	S	R	R	R	S	S	R	S	S	S	S	S	S	S
30	R	R	S	R	R	R	S	S	R	S	S	S	S	S	S	S
31	R	R	S	S	R	R	S	S	R	R	S	S	S	S	S	S
32	R	R	S	R	R	R	S	S	R	S	S	S	S	S	S	S
33	R	R	R	R	R	R	S	S	S	S	S	S	S	S	S	S
34	R	R	R	R	R	R	S	S	S	S	S	S	S	S	S	S
35	R	R	S	R	R	R	S	R	S	S	S	S	S	S	S	S
36	R	R	S	R	R	R	S	S	R	S	S	S	S	S	S	S
37	R	R	S	R	R	R	S	S	R	S	S	S	S	S	S	S
38	R	R	S	R	R	R	S	S	S	S	S	S	S	S	S	R
39	R	R	R	R	S	S	S	S	S	R	R	S	S	S	S	S
40	R	R	R	R	R	R	S	S	S	S	S	S	S	S	S	R
41	R	R	S	R	R	R	S	S	R	R	S	S	S	S	S	S
42	R	R	S	R	R	R	S	S	R	R	S	S	S	S	S	S
43	R	R	R	S	R	R	S	S	R	R	R	S	S	S	S	S
44	R	R	R	S	R	R	S	S	R	R	R	S	S	S	S	S
45	R	R	R	R	R	R	S	S	S	S	S	S	S	R	S	R
46	R	R	R	R	R	R	S	S	S	S	S	S	S	R	S	R
47	R	R	R	R	R	R	S	S	S	S	S	S	S	R	S	R

48	R	R	R	R	R	R	S	S	R	S	S	S	S	S	S	R
49	R	R	R	R	R	R	S	S	S	S	S	S	S	R	S	R
50	R	R	S	R	R	R	R	R	R	R	S	S	S	S	S	R
51	S	S	S	S	S	S	S	S	S	S	S	S	S	S	S	S
52	S	S	S	S	S	S	S	S	S	S	S	S	S	S	S	S
53	S	S	S	S	S	S	S	S	S	S	S	S	S	S	S	S
54	S	S	S	S	S	S	S	S	S	S	S	S	S	S	S	S
55	S	S	S	S	S	S	S	S	S	S	S	S	S	S	S	S
56	S	S	S	S	S	S	S	S	S	S	S	S	S	S	S	S
57	S	S	S	S	S	S	S	S	S	S	S	S	S	S	S	S
58	S	S	S	S	S	S	S	S	S	S	S	S	S	S	S	S
59	S	S	S	S	S	S	S	S	S	S	S	S	S	S	S	S
60	S	S	S	S	S	S	S	S	S	S	S	S	S	S	S	S
61	S	S	S	S	S	S	S	S	S	S	S	S	S	S	S	S
62	S	S	S	S	S	S	S	S	S	S	S	S	S	S	S	S
63	S	S	S	S	S	S	S	S	S	S	S	S	S	S	S	S
64	S	S	S	S	S	S	S	S	S	I	S	S	S	S	S	S
65	S	S	S	S	S	S	S	S	S	I	S	S	S	S	S	S
66	S	S	S	S	S	S	S	S	S	I	S	S	S	S	S	S
67	S	S	S	S	S	S	S	S	S	I	S	S	S	S	S	S
68	S	S	S	S	S	S	S	S	S	I	S	S	S	S	S	S
69	S	S	S	S	S	S	S	S	S	I	S	S	S	S	S	S
70	S	S	S	S	S	S	S	S	S	I	S	S	S	S	S	S
71	S	S	S	S	S	S	S	S	S	I	S	S	S	S	S	S
72	S	S	S	S	S	S	S	S	S	I	S	S	S	S	S	S
73	S	S	S	S	S	S	S	S	S	I	S	S	S	S	S	S
74	S	S	S	S	S	S	S	S	S	I	S	S	S	S	S	S
75	S	S	S	S	R	S	S	S	S	S	S	S	S	S	S	S
76	S	S	S	S	S	S	S	S	S	S	S	S	S	S	S	R
77	S	S	S	R	S	S	S	S	S	I	S	S	S	S	S	S
78	S	S	S	R	S	S	S	S	S	I	S	S	S	S	S	S
79	S	S	S	R	S	S	S	S	S	I	S	S	S	S	S	S
80	S	S	S	R	S	S	S	S	S	I	S	S	S	S	S	S
81	S	S	R	S	S	S	S	S	S	R	S	S	S	S	S	S
82	S	S	S	S	S	S	S	S	R	R	S	S	S	S	S	S
83	S	S	S	S	R	R	S	S	S	S	S	S	S	S	S	S

84	S	S	S	S	S	S	S	S	R	R	S	S	S	S	S	S
85	S	S	S	S	S	S	S	S	R	R	S	S	S	S	S	S
86	S	S	S	S	S	S	S	S	R	R	S	S	S	S	S	S
87	S	S	S	S	R	R	S	S	S	S	S	S	S	S	S	S
88	S	S	S	S	R	R	S	S	S	S	S	S	S	S	S	S
89	S	S	S	S	S	S	S	S	R	R	S	S	S	S	S	S
90	S	S	S	S	S	S	S	S	R	R	S	S	S	S	S	S
91	S	S	R	R	S	S	S	S	S	I	S	S	S	S	S	S
92	S	S	R	R	S	S	S	S	S	I	S	S	S	S	S	S
93	S	S	R	R	S	S	S	S	S	S	S	S	S	S	S	R
94	S	S	S	S	S	S	S	S	S	R	R	S	S	S	S	R
95	S	S	S	S	S	S	S	S	R	R	S	S	S	R	S	S
96	S	S	S	R	R	R	S	S	S	S	R	S	S	S	S	S
97	S	S	S	R	R	R	S	S	S	S	R	S	S	S	S	S
98	S	S	R	R	R	R	S	S	S	I	S	S	S	S	S	S
99	S	S	S	R	R	R	S	S	R	I	S	S	S	S	S	S
100	S	S	R	R	R	R	S	S	R	R	R	S	S	S	R	S

***Supplementary Table S3**. Antibiotic resistance profiles of each S. aureus isolate tested. Isolates numbered from 1 to 50 are MRSA strains and isolates numbered from 51 to 100 are MSSA strains. S stands for sensitive to that antibiotic (green), R for resistant (red) and I for intermediate (yellow). MET: methicillin, OXA: oxacillin, GEN: gentamicin, TOB: tobramycin, CIP: ciprofloxacin, LEV: levofloxacin, TMX: trimetrexate, FOS: fosfomycin, ERY: erythromycin, CLI: clindamycin, TET: tetracycline, VAN: vancomycin, TEI: teicoplanin, DAP: daptomycin, LIN: linezolid, MUP: mupirocin.*

GLMR results for CS

	**coef**	**std err**	**z**	***p***	**[0.025**	**0.975]**
**Intercept**	40.081	5.261	7.618	0.000	29.769	50.393
**MET (R)**	-17.244	7.044	-2.448	0.014*	-31.051	-3.437
**OXA (R)**	-17.244	7.044	-2.448	0.014*	-31.051	-3.437
**GEN (R)**	2.285	16.165	0.141	0.888	-29.397	33.967
**TOB (R)**	8.241	12.613	0.653	0.514	-16.481	32.962
**CIP (I)**	-48.101	38.211	-1.259	0.208	-122.993	26.790
**CIP (R)**	134.039	45.851	2.923	0.003**	44.173	223.904
**LEV (I)**	-169.349	72.919	-2.322	0.020*	-312.267	-26.431
**LEV (R)**	-127.759	47.217	-2.706	0.007**	-220.302	-35.215
**TMX (R)**	-39.575	60.793	-0.651	0.515	-158.727	79.578
**FOS (R)**	4.931	33.082	0.149	0.882	-59.909	69.770
**ERY (R)**	7.495	15.722	0.477	0.634	-23.318	38.309
**CLI (I)**	-14.198	15.599	-0.910	0.363	-44.771	16.375

**CLI (R)**	-10.825	19.693	-0.550	0.583	-49.422	27.773
**TET (R)**	12.884	18.845	0.684	0.494	-24.052	49.819
**VAN (I)**	-13.669	46.866	-0.292	0.771	-105.525	78.187
**TEI (S)**	40.081	5.261	7.618	0.000***	29.769	50.393
**DAP (R)**	-9.800	25.153	-0.390	0.697	-59.099	39.500
**LIN (R)**	24.678	50.909	0.485	0.628	-75.103	124.458
**MUP (R)**	-16.059	19.772	-0.812	0.417	-54.812	22.693

Supplementary Table S4. *GLMR results for MIC_{50} values of the CS extract against all S. aureus isolates. MET: methicillin, OXA: oxacillin, GEN: gentamicin, TOB: tobramycin, CIP: ciprofloxacin, LEV: levofloxacin, TMX: trimetrexate, FOS: fosfomycin, ERY: erythromycin, CLI: clindamycin, TET: tetracycline, VAN: vancomycin, TEI: teicoplanin, DAP: daptomycin, LIN: linezolid, MUP: mupirocin. (R), (I) and (S) indicate resistant, intermediate and sensitive, respectively. Gray shaded rows indicate significant values (p <0.05, *; p <0.01, **; p <0.001, ***).*

GLMR results for GP

	**coef**	**std err**	**z**	***p***	**[0.025**	**0.975]**
**Intercept**	27.835	6.668	4.174	0.000	14.765	40.904
**MET (R)**	4.541	9.157	0.496	0.620	-13.407	22.489
**OXA (R)**	4.541	9.157	0.496	0.620	-13.407	22.489
**GEN (R)**	12.682	20.970	0.605	0.545	-28.418	53.781
**TOB (R)**	-6.177	16.418	-0.376	0.707	-38.355	26.002
**CIP (I)**	-48.769	49.565	-0.984	0.325	-145.914	48.376
**CIP (R)**	31.607	27.539	1.148	0.251	-22.369	85.583
**LEV (I)**	57.463	48.239	1.191	0.234	-37.084	152.009
**LEV (R)**	-25.856	24.456	-1.057	0.290	-73.788	22.077
**TMX (R)**	27.004	78.893	0.342	0.732	-127.623	181.632
**FOS (R)**	-52.315	42.908	-1.219	0.223	-136.414	31.783
**ERY (R)**	37.831	20.421	1.853	0.064	-2.193	77.855
**CLI (I)**	-13.378	20.463	-0.654	0.513	-53.485	26.728
**CLI (R)**	-38.594	25.609	-1.507	0.132	-88.786	11.598
**TET (R)**	22.866	24.542	0.932	0.351	-25.235	70.967
**VAN (I)**	-44.631	60.765	-0.734	0.463	-163.729	74.466
**TEI (S)**	27.835	6.668	4.174	0.000***	14.765	40.904
**DAP (R)**	4.739	32.627	0.145	0.885	-59.208	68.687
**LIN (R)**	-56.900	66.111	-0.861	0.389	-186.474	72.675
**MUP (R)**	-3.588	25.625	-0.140	0.889	-53.813	46.636

Supplementary Table S5. *GLMR results for MIC_{50} values of the GP extract against all S. aureus isolates. MET: methicillin, OXA: oxacillin, GEN: gentamicin, TOB: tobramycin, CIP: ciprofloxacin, LEV: levofloxacin, TMX: trimetrexate, FOS: fosfomycin, ERY: erythromycin, CLI: clindamycin, TET: tetracycline, VAN: vancomycin, TEI: teicoplanin, DAP: daptomycin, LIN: linezolid, MUP: mupirocin. (R), (I) and (S) indicate resistant, intermediate and sensitive, respectively. Gray shaded rows indicate significant values (p <0.001, ***).*
